# The complete mitochondrial genome of *Helophilus virgatus* (Diptera: Syrphidae: Eristalinae) with a phylogenetic analysis of Syrphidae

**DOI:** 10.1080/23802359.2019.1667890

**Published:** 2019-09-19

**Authors:** Hu Li, Juan Li

**Affiliations:** a College of Life Sciences, Northwest University, Xi’an, Shaanxi, China;; b Shaanxi Key Laboratory of Bio-resources, School of Biological Science & Engineering, Shaanxi University of Technology, Hanzhong,Shaanxi, China

**Keywords:** Syrphidae, mitochondrial genome, phylogeny

## Abstract

The complete mitochondrial genome (mitogenome) of *Helophilus virgatus* (Coquilletti, 1898) was sequenced. Its whole mitogenome was 15,742 bp in length, and all 37 genes were in the ancestral gene arrangement. ATN was used as start codon in most of PCGs except for *ND1* and *COX1*, which used TTG. Besides, *ATP8*, *ND3*, *ND5*, and *ND6* ended with an incomplete T, the others used TAA as termination codons. A phylogenetic tree based on 13 PCGs from 14 species (11 Syrphidae and 3 outgroup species) shows that Syrphinae and Eristalinae form a sister group, and supports the monophyly of Syrphidae.

*Helophilus virgatus* (Coquilletti, 1898), a very common hoverfly sharing a loop in vein R_4 + 5_ on wings, a thorax that has pale, longitudinal stripes on the top, and abdomen with yellow maculae only on dorsal surface of the second segment, belongs to the tribe Eristalini of subfamily Eristalinae (Diptera: Syrphidae). The adults have a habit of visiting flowers but their larvae are largely saprophagous and feed on dead or decaying plants including sap runs, rotting wood, rot-holes, and rotting vegetation (Huo et al. 2007; Huang and Cheng 2012; Ball and Morris 2015).

So far, only 11 syrphine species’ mitogenomes have been sequenced completely or partially and are available in the GenBank (https://www.ncbi.nlm.nih.gov/) (Cameron et al. 2007; Tang et al. 2014; Junqueira et al. 2016; Li et al. 2017; Pu et al. 2017; Sonet et al. 2019). Herein, we sequenced the twelfth mitogenome, of *H*. *virgatus*, the detailed information about this sequence and a phylogenetic tree based on dataset of all syrphine mitogenomes are given in the following text.

Specimens of *H*. *virgatus* were collected in a rape field of Leijiaxiang Village (107°03'E, 33°04'N) in Hanzhong City of Shaanxi Province, China in March 2019. The samples were immediately immersed in absolute ethanol and frozen in −20 °C in the Museum of Zoology and Botany, Shaanxi University of Technology, Hanzhong, China (SUHC) where the specimen and its DNA are stored (accession number of the specimen for sequencing in this study is 201901-25-1). Genomic DNA was extracted using a DNeasy^©^ Tissue Kit (Qiagen, Hilden, Germany), and mitogenome was sequenced by the next-generation sequencing (Illumina HiSeq 2500, 2 Gb raw data; Berry Genomic, Beijing, China), as well as, PCR amplification.

The whole length of the complete mitogenome of *H*. *virgatus* was 15,742 bp (GenBank No.: MN148445) containing 37 genes including 22 transfer RNA genes, 13 protein-coding genes (PCGs), 2 ribosomal RNA genes, and a non-coding region, the pattern of mitogenome genes in the newly sequenced species was similar to other Syrphidae (Clary and Wolstenholme 1985; Li et al. 2017; Pu et al. 2017), of which 23 genes were encoded in J-strand, 14 genes were located in N-strand.

The whole nucleotide composition of the mitogenome of *H*. *virgatus* was 40.9% of A, 38.8% of T, 8.5% of G, and 11.8% of C, respectively, and showed a high A + T bias of 79.7%. Most PCGs started with ATN codon, expect *ND1* and *COX1*, which started with TTG codon. Most ended with TAA codon, but *ATP8*, *ND3*, *ND5*, and *ND6* ended with an incomplete T codon.

All of the genes were matched to *Drosophila melanogaster*. The gene intergenic and overlapping region included 14 gene intervals with the maximum of 26 bp between *tRNA-Glu* and *tRNA-Phe*, and 10 overlaps with maximum of 9 bp among *tRNA-Trp* and *tRNA-Cys*. But the gene rearrangement was not exposed in this mitogenome.

A phylogenetic tree was constructed based on the concatenated dataset of 13 PCGs from 14 Diptera species including 11 Syrphidae and 3 outgroup species by the method of maximum-likelihood (ML) (Figure 1), in which 13 PCGs were obtained from GenBank, *Nemopoda mamaevi*, *Megasalia scalaris*, and *Fergusonina taylori* were used as outgroups. The result clearly shows that *H. virgatus* clustered with the clade of Eristalinae and was close to *Eristalis* and *Eristalinus* genera, both of which belonged to the tribe Eristalini. Syrphinae and Eristalinae formed the sister groups, and highly supported the monophyly of Syrphidae, which was consistent with other studies (Pu et al. 2017; Pauli et al. 2018).

**Figure 1.**
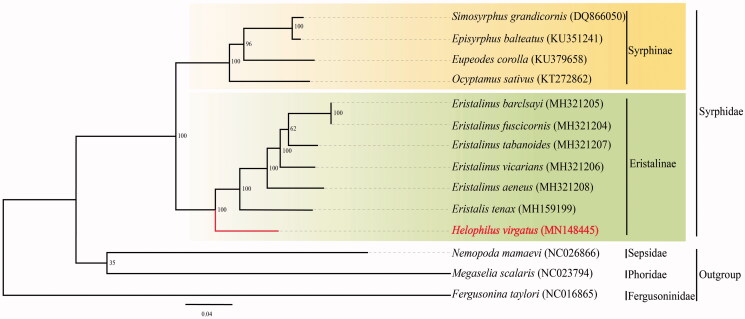
Phylogenetic tree of 14 Diptera species based on the concatenated dataset of 13 PCGs using the maximum-likelihood (ML) method. GenBank accession Numbers are shown in the brackets after each species name.
